# Targeted Combination of Poly(ADP-ribose) Polymerase Inhibitors and Immune Checkpoint Inhibitors Lacking Evidence of Benefit: Focus in Ovarian Cancer

**DOI:** 10.3390/ijms25063173

**Published:** 2024-03-09

**Authors:** Morgan Bailey, Susan Morand, Rachel Royfman, Leslie Lin, Aditi Singh, Laura Stanbery, Adam Walter, Danae Hamouda, John Nemunaitis

**Affiliations:** 1 Department of Medicine, University of Toledo, Toledo, OH 43614, USA; 2 Gradalis, Inc., Dallas, TX 75006, USA; lnejedlik@gradalisinc.com (L.S.);; 3 Department of Gynecologic Oncology, Promedica, Toledo, OH 43614, USA

**Keywords:** ovarian cancer, PARP inhibitor, immunotherapy, BRCA, homologous recombination, HRD, HRP, checkpoint inhibitor

## Abstract

The emergence of targeted therapeutics in ovarian cancer, particularly poly (ADP-ribose) polymerase inhibitors (PARPi’s), has created additional opportunities for patients seeking frontline and recurrent disease management options. In particular, PARPi’s have shown clinical benefits in BRCA mutant and/or homologous recombination deficient (HRD) ovarian cancer. Until recently, response was thought to be limited in *BRCA* wild-type, homologous recombination proficient (HRP) cancers. Therefore, attempts have been made at combination therapy involving PARPi to improve patient outcomes. Additionally, immune checkpoint inhibitors (ICIs) have demonstrated underwhelming results involving ovarian cancer. Many are searching for reliable biomarkers of immune response to increase efficacy of ICI therapy involving ovarian cancer. In this review, we examine the evidence supporting the combination of PARPi and ICIs in ovarian cancer, which is still lacking.

## 1. Introduction

The successful application of molecular-based and pathway-directed technology has shifted the focus of anticancer drug development toward precision medicine, where novel therapeutics have met success in the clinical sphere [1]. A molecular approach that has gained traction in gynecologic oncology is the inhibition of the poly(ADP-ribose) polymerase (PARP) family of proteins, which are centrally involved in DNA repair [2,3,4,5]. Early use of these therapeutics was focused on cells with DNA repair deficiency, initially represented by somatic or germline mutations involving *BRCA1* and/or *BRCA2* genes. In gynecologic oncology, PARPi’s were initially tested in ovarian cancer, which has a 15–30% prevalence of somatic or germline *BRCA1/2* mutations [4]. Recently, their application has been expanded to include multiple cancers and has extended beyond *BRCA1/2* mutations containing tumors to include a broader range of genetic mutations, involving DNA repair and defects involving homologous repair pathway [4,6], such as *BRIP1*, *RAD51C*, *RAD51D*, *ATM*, *PALB2*, *CHEK2*, etc.

Improvements in clinical outcomes have also been found in the field of cancer immunotherapy, particularly with checkpoint inhibitors [7]. In other cancers, anticancer activity of checkpoint inhibitors has also been demonstrated as enhanced via the expression of various biomarkers, including programmed death ligand-1 (PD-L1), tumor mutational burden (TMB), tumor-infiltrating lymphocytes (TILs), and components of the tumor microenvironment (TME) [8,9,10]. However, the application of immunotherapies in ovarian cancers has not shown significant clinical benefit compared to standard of care, with trials such as IMagyn050/GoG failing to demonstrate benefits in the primary outcome of PFS (progression-free survival) [11,12,13]. Potential exploration for a subset population more sensitive to ICIs may require strategies which include the careful selection of patients based on biomarker/molecular profile guidance and/or involvement of relevant combination therapy [13]. Herein, we will review the mechanism and rationale for the application of PARPi and immune checkpoint therapy along with the results of clinical trials examining this combination.

## 2. PARPi Mechanism

PARP proteins are a class of 17 nucleoproteins that utilize nicotinamide adenine dinucleotide (NAD+) as a cofactor to transfer one or several ADP-ribose group(s) using ADP-ribosyl transferase (ART) at their catalytic site [3]. Poly-ADP-ribosylation (PARylation) refers to the post-translational addition of a poly(ADP-ribose) group to a protein [2,3,14]. In PARylation, negatively charged PARs are covalently bound to glutamate, aspartate, or lysine residues of target proteins. PARylation regulates the functions and interactions of proteins, induces protein degradation by proteasomes, and (de)stabilizes the interactions of proteins with DNA [15]. The interaction of proteins with DNA is an essential process for DNA repair. The auto-PARylation of PARP1/2 results in dissociation from DNA, a crucial advancement that permits the repair enzymes recruited by PARP1/2 to access the single broken DNA strand [16]. PARP’s central role in protein modification makes it essential for the conformational changes required for DNA repair machinery to aggregate as a unit. PARP proteins have distinct functions and only PARP1/2/3/4/5a/5b possess the potential to activate PARylation [17]. PARP1/2 are essential for DNA repair and catalyze long PAR chains [15]. PARP1 is thought to perform 90% of PARylation involved in DNA damage repair, including activity in base excision repair, nucleotide excision repair, homologous recombination (HR), non-homologous end joining (NHEJ), and alternative NHEJ [15,17]. PARP1–3 have been proven to play a role in base excision repair [2].

Capitalizing on the role of PARP in DNA repair, PARPi have been developed as an anticancer therapy to destabilize DNA repair machinery in cancer cells. PARPi compete with NAD+ at the PARP catalytic domain to trap PARP-DNA complexes on endogenous DNA breaks, thereby damaging DNA and interfering with replication [15,16,17]. PARPi’s specifically target malignant cells with defective DNA repair to induce cell death via synthetic lethality. Synthetic lethality refers to a phenomenon when multiple deficiencies, in this case inhibiting DNA repair, results in cell death. Consequently, without DNA repair mechanisms, DNA damage accumulates and the cell is unable to maintain normal survival and replication functions, resulting in cellular death [15].

### Clinical Evidence of PARPi Therapy in Ovarian Cancer

The efficacy and therapeutic benefits seen with PARPi’s in the treatment and maintenance management of ovarian cancer has been demonstrated by multiple randomized, double-blind, multicenter studies leading to its eventual introduction in clinical practice [18,19,20]. Currently, olaparib, niraparib, and rucaparib are approved for clinical use in the treatment of ovarian cancer by the U.S. Food and Drug Administration (FDA). Veliparib, although not FDA approved, has been granted Orphan Drug Status.

Olaparib is FDA approved for use with frontline BRCA mutated ovarian cancer following adjuvant platinum based chemotherapy. SOLO1, with twice daily olaparib, demonstrated a significant 70% reduction in death or progression at 3 years [20]. Five-year follow up data from that trial maintained a significant advantage in PFS, HR 0.33 [18]. PAOLA-1 combined olaparib with bevacizumab in frontline ovarian cancer patients, and a significant PFS advantage was noted in the *BRCA*m (HR 0.33) and HRD (HR 0.43) subpopulations but no benefit was identified in the HRP subpopulation (HR 1.0) [21]. olaparib is also indicated in *BRCA* mutated second line platinum sensitive recurrence, with a PFS benefit of 19.1 mos vs. 5.5 mos [22]. Olaparib had previously been approved in the 3rd line or later setting for *BRCA*m; however, the indication was voluntarily withdrawn (26 August 2022) due to concerns regarding the OS data favoring control.

Niraparib is approved for front line maintenance therapy of patients with epithelial ovarian, fallopian tube, or primary peritoneal cancer, following chemotherapy. The PRIMA trial evaluated all patients independent of *BRCA* or HRD status and a significant benefit in PFS 13.8 mos vs. 8.2 mos was observed. When patients were evaluated by HRD status the patients with HRD tumors experienced benefit with improvement in PFS of 21.9 mos vs. 10.4 mos [19]. Patients with HRP tumors had minimal benefit in PFS of only 2.7 months (HR 0.68) which limits clinical use in that population given ≥Grade 3 product related toxic effect of 60–70% (thrombocytopenia, anemia, neutropenia), 69–80% dose interruption due to toxicity risk and 1–2% risk of treatment induced AML/MDS [19,23]. Niraparib has been studied in the NOVA trial leading to initial approval in all patients with platinum sensitive recurrence who experienced partial or complete response [24]. Following concerns regarding the overall survival (OS) data in non-g*BRCA* patients (8 December 2022), Niraparib is now approved only in g*BRCA* mutated platinum sensitive recurrences (11 November 2022) [25]. Niraparib was voluntarily withdrawn from the fourth line or greater setting (14 September 2022) due to the inability to define safety from an OS perspective [26].

Rucaparib is used as a single-agent treatment for recurrent *gBRCA* or *sBRCA* mutated ovarian cancer relapsed after at least two lines of chemotherapy and maintenance treatment of recurrent epithelial ovarian, fallopian tube, or primary peritoneal cancers that are in complete or partial response to platinum-based chemotherapy. ARIEL2 was a Phase II study that demonstrated significant reduction in risk of recurrent ovarian cancer progression during rucaparib treatment in the *BRCA* mutated subgroup (12.8 months; HR 0.27, *p* < 0.0001) and the wild-type *BRCA* with high loss of heterozygosity subgroup (HRP, 5.7 months; HR 0.62, *p* = 0.011) when compared to the wild-type *BRCA* with low loss of heterozygosity subgroup (5.2 months) [27]. ARIEL3 was multicenter Phase III study that evaluated rucaparib as maintenance treatment for *BRCA* mutated carcinomas and HRD-positive tumors. Patients with *BRCA* mutated tumors who were treated with rucaparib had a median PFS of 16.6 months compared to 5.4 months with placebo (HR 0.23, *p* < 0.0001), while PFS in HRD-positive tumors treated with rucaparib was 13.6 months versus 5.4 months in the placebo group (HR 032, *p* < 0.0001) [28]. Rucaparib was also originally approved in fourth line treatment for *BRCA*m based on PFS date from ARIEL4. There were again significant concerns regarding OS in this population and the indication was voluntarily withdrawn (10 June 2022).

The FDA has granted veliparib Orphan Drug Designation for treatment of epithelial ovarian cancer in combination with DNA-damaging agents. VELIA/GOG-3005 was an international, Phase III, placebo-controlled trial that demonstrated significantly increased PFS in patients with stage III or IV high-grade serous ovarian carcinoma treated with a regimen of veliparib with induction chemotherapy and veliparib maintenance therapy. In the *BRCA* mutation cohort, the median PFS was 34.7 months in the group treated with carboplatin, paclitaxel, and veliparib induction therapy, followed by veliparib maintenance therapy, and 22.0 months in the group treated with carboplatin plus paclitaxel induction therapy alone (HR 0.44, *p* < 0.001) [29]. The median PFS in the HRD cohort was 31.9 months and 20.5 months, respectively (HR 0.57, *p* < 0.001); 23.5 months and 17.3 months in the intention-to-treat population (HR 0.68, *p* < 0.001) [29].

However, recently FDA has been undergoing review of late stage patients (≥2 prior chemotherapy regimens) who have taken PARPi’s to assess OS benefit (Table 1). Specifically, review of SOLO3 (olaparib), Ariel4 (rucaparib), Quadra and Nova (niraparib) studies. Recent communications to health care providers on 26 August 2022 for olaparib [30], 10 June 2022 for rucaparib [31] and 14 September 2022 and 11 November 2022 [25,26] for niraparib were issued following multiple discussions with FDA. Discussions with pharmaceutical sponsors revealed concerns regarding OS compromise in patients receiving PARPi’s following ≥2 prior lines of chemotherapy. These results suggest the detrimental long term effect on OS and led to the reclassification of ASCO guidelines restrict the use of PARPi treatment in late stage ovarian cancer on 27 September 2022 [32].

## 3. Biomarkers

Several biomarkers have been associated with increased PARPi sensitivity, with the most widely studied being *BRCA1/2* and the homologous repair pathway. Prior publications have shown that PARP inhibition promoted synthetic lethality in *BRCA1/2* mutant cancers via inhibition of single strand break repair and base excision repair pathways [2]. Treatment with a PARPi in *BRCA1* mutant cancers causes diminished heterodimerization with BRCA1 associated ring domain 1 (BARD1) and has been shown to decrease PAR formation and recruitment of BRCA1/BARD1 complex to the site of DNA damage. This impairment results in the consequent inhibition of HR mediated repair and apoptosis [15]. Finally, in cases of defective NHEJ and HR, PARP1 inhibition led to malignant cell apoptosis by avoiding alternate end joining activation [33].

## 4. Immune Activity

PARPi therapy leads to the accumulation of cytoplasmic DNA, which activates the cGAS/STING pathway, thereby creating an influx of natural killer (NK) cells, T-lymphocytes, and tumor-infiltrating lymphocytes (TIL) within the TME [34,35]. PARPi also enhances the suppressive function of regulatory T-cells (Treg), which facilitates anticancer immune-mediated activity [36].

Other reports suggest an opposite effect on immune response that renders cancer cells more resistant to T-cell mediated death. PARPi therapy has been shown to exert immunosuppressive effects by upregulating PD-L1 expression on the surface of cancer cells. Binding between PD-L1 and PD-1 receptors on activated T-cells leads to subsequent attenuation of T-cell killing by suppressing T-cell proliferation and cytokine release [37]. Evidence of combination ICI and PARPi treatment enhancing immune activity has been suggested to be partly related to PARPi-mediated disruption of DNA repair and generation of DNA damage with a subsequent increase in neoantigen expression, which theoretically creates a more sensitive and favorable signal response (high TMB) to ICIs [38,39,40]. Therefore, PARPi may act synergistically with ICIs by priming the tumor microenvironment [41]. However, this theoretical increase in ICI sensitivity is tempered by the fact that the resultant neoantigens are likely subclonal rather than clonal [42]. McGranahan et al. demonstrated that clonal neoantigens are required for optimal sensitivity to ICIs, therefore, an increase in subclonal neoantigens may decrease sensitivity to ICIs [43].

Limitations of PARPi treatment include its limited benefit in ovarian cancer patients, coupled with a toxic AE profile. Most common toxicities are hematological-related, as well as interference with T-cell homeostasis and maturation of CD8^+^ T cells [44,45]. Moreover, ovarian cancer cells often have defective STING signaling and are unable to respond to PARPi-mediated STING activation [46]. Finally, PARPi has been shown to induce treatment related acute myeloid leukemia and myelodysplastic syndrome in a small population of patients, further limiting a robust immune response.

A useful biomarker predictive of PARPi sensitivity is cyclin-dependent kinase 12 (CDK12). CDK12 regulates transcriptional elongation of genes by phosphorylating RNA polymerase II via complexing with cyclin K. It is also involved in the progression through the cell cycle, RNA splicing, cell proliferation, maintenance of genomic stability, and response to DNA damage. A mutation in, or lack of, CDK12 results in PARPi sensitization [47]. The combination of PARPi and CDK12 inhibitors has also been highly active in many models of Ewing’s sarcoma and high grade serous ovarian cancer [15,47,48]. Johnson et al. studied dinaciclib as an inhibitor of CDK12 in triple negative breast cancer (TNBC) stratified by *BRCA* status and prior response to PARPi using patient derived xenograft models. The *BRCA* wild-type population showed reduced HR expression following treatment with dinaciclib, which was associated with sensitization to PARP inhibition by reducing the IC_50_. In the *BRCA* mutant population, resistance to PARPi was lessened when dinaciclib was added. This was demonstrated using a model derived from a TNBC patient with a germline S1970* *BRCA2* mutation. The combination of dinaciclib and PARPi resulted in tumor growth inhibition lasting at least 60 days compared to about 40 days with cisplatin and a PARPi. Finally, in the *BRCA* mutant population who had achieved control of cancer growth with PARPi monotherapy, the addition of CDK12 inhibition demonstrated prolonged tumor regression [49].

PARP enzymes may play a role in the recruitment of NK cells, neutrophils, macrophages, and dendritic cells (DCs) [50]. PARPi has been shown to augment transcription within macrophages via LPS-induced proinflammatory genes such as TNF-α, IL-1, and IL-6 [50]. Additionally, PARP-1 stimulates the production of CCL2, of which the CCL2-CCR2 axis plays a vital role in NK cell recruitment to sites of infection [51]. Moreover, they modify the adaptive immune response by modulating DC ability to stimulate T and B cell differentiation [52]. This is accomplished via PARP-1 activation of nuclear factor-κB and nuclear factor-AT which promotes various adhesion molecule, chemokine, and cytokine expression across many immune effector cell types. PARPi modulates T-cell expression of IL-2 while simultaneously inhibiting expression of Foxp3 cells [52]. Tumor cells that avoid the initial immune response may have acquired immune tolerance via either loss of human leukocyte antigens (HLA) or generation of an immunosuppressive microenvironment by downregulating Tregs, macrophages, checkpoint molecules (PD-L1), deprivation of nutrients and oxygen, and release of immunosuppressive cytokines [50]. Current literature suggests that PARPi may alter how tumor cells evade the immune response by either causing direct cancer cell cytotoxicity or alternative immuno-stimulation (cGAS/STING pathways, paracrine stimulation of DCs, and increased T-cell infiltration) [53], although, as described above, cancers with high population of defective STING signals may be less responsive (i.e., ovarian cancer).

### PARPi Relationship to Interferons/Chemoattractants

PARPi plays a role in immunomodulation through type 1 interferons (IFNs) via the cyclic GMP-AMP synthase and stimulator of interferon genes (cGAS-STING) pathway [54]. IFNs are pro-inflammatory cytokines responsible for defense against foreign pathogens such as viruses and bacteria through the innate and adaptive immune responses [55]. cGAS-STING signaling promotes the release of type 1 IFNs to provide antimicrobial and antitumor immunity [56]. In in vitro and murine models, the use of PARPi caused an accumulation of cytosolic DNA fragments, which stimulated the cGAS-STING pathway to release type 1 IFNs capable of inducing an immune response independent of *BRCA* status. The same study then investigated PARPi combined with ICIs, which further enhanced the impact of PARPi in murine models [54]. This represents a mechanistic link between PARPi and ICIs through PARPi-driven immunomodulation.

In *BRCA1*-deficient TNBC, olaparib treatment increased tumor infiltration and CD8^+^ T cell activation in vivo through the cGAS/STING pathway as well. Veliparib and talazoparib had the same effect, suggesting that cGAS/STING activation may occur with other PARPi’s [57]. When stratified by HR status, the recruitment of T-cells in in vivo models was found to be more prominent in HRD compared to HRP [57]. Also, an increase in chemoattractants targeting CD8^+^ T-cells (CCL5 and CXCL10) was higher in HRP cells compared to HRD cells following PARPi therapy, whereas IFN-beta was exclusively induced by olaparib in the HRD cells [57]. This suggests that CCL5 and CXCL10 may be STING-independent, while IFN is more strongly linked to the PARPi-induced cGAS/STING pathway in the HRD group. In patients with defective Excision Repair Cross-Complementation Group 1 (ERCC1), a particular homologous repair deficiency, with non-small cell lung cancer (NSCLC), PARPi therapy created cytoplasmic chromatin fragments that triggered cGAS/STING to produce type I IFNs and CCL5 [58]. The impact of PARPi on chemokine induction in DNA repair proficient individuals may be less effective than in DNA deficient individuals.

## 5. PARPi on CD4^+^ Treg Cells

T-cell development is a highly coordinated process which involves several steps of maturation, multiple transcription factors, and cytokines [53]. PARPi can pharmacologically impact the T-cell compartment and transiently alter cell immune response by removing PARP from the signaling cascade [50]. For example, PARPi causes increased Nuclear Factor of Activated T-cells (NFAT) activation, increased NF-κB activation, and reduced IL-2 response [59]. Both NFAT and NF-κB are important transcription factors that promote development and activation of various immune cells, while IL-2 is a pro-T-cell cytokine [60,61,62]. One class of immune cells impacted by PARP proteins includes Tregs, which function to negatively regulate the immune response [50]. An important transcription factor for CD4^+^ Tregs is forkhead box p3 (Foxp3^+^); overexpression of Foxp3^+^ results in increased cellular proliferation, migration, and invasion of Tregs [63]. Additionally, increased expression of Foxp3^+^ induces expression of pro-apoptotic genes including *PARP* [64]. Intact PARP-1 protein downregulates the suppressive function of Tregs by decreasing expression of Foxp3^+^ mRNA [50]. The inverse has been studied; in *PARP* knock-out mice, greater frequencies of Foxp3^+^ were expressed, resulting in increased number of inducible Tregs (iTregs), which are active in immunosuppression [59]. An additional study found that pharmacological inhibition of PARP-1 caused Foxp3^+^ stabilization, consequent upregulation of genes downstream from Foxp3^+^, and improved immunosuppressive function of Tregs [36]. In sum, PARPi stabilizes and enhances Treg, thereby promoting immune suppression [36].

### 5.1. PARPi Interferes with DC Maturation and Recruitment

DCs are specialized antigen presenting cells (APC) critical for T-cell self-versus non-self-recognition and T-cell activation [50]. The PARP enzyme aids in DC differentiation and development from monocytes [65]. Upon interaction with antigens, DCs begin maturation by triggering migration and upregulation of CD80^+^ and CD86^+^ molecules to cell surface [66]. When appropriate levels of both CD80^+^ and CD86^+^ molecules are achieved, DCs will begin to migrate with CD83^+^ molecules signaling a completion in the maturation process [66]. As such, CD83^+^ is considered a mature DC-specific marker [66]. Upon interaction with PARPi, fewer DC cells exhibit CD86^+^, with no changes to CD80^+^ [66]. Given that maturation is incomplete without CD86^+^, fewer DC cells express CD83^+^. Moreover, PARPi also reduces IL-12, IL-10, and several other cytokines which further affects the functional maturation of DC [66]. The overall effect of PARPi on DC cells is dose-dependent, where at higher doses DC maturation and recruitment is further reduced [66].

### 5.2. PARPi Effect on T Cell Homeostasis and Maturation

The PARP enzyme helps regulate genetically induced activation of neutrophils, macrophages, DC cells, microglia, and the pro-inflammatory response. Given their broad range of action, PARP enzymes play a central role in maintaining homeostasis via Treg cells [52,59]. PARPi interferes with this process by altering maturation and increasing the quantity of Treg cells [50,59]. Moreover, PARPi inhibits the expression of pro-inflammatory cytokines such as TNF-α, IL-1, and IL-6 by macrophages [50]. This alteration changes the microenvironment by damaging macrophagic function and results in the accumulation of free radical species, cellular debris, and potential infective agents [50]. A combination of these effects can result in oxidative stress which may induce apoptosis of CD8^+^ T cells and NK cells [52]. The reduction in the cytotoxic arm of both the innate and adaptive immune systems can thus be exploited by tumorigenic cells to evade immune response.

### 5.3. PARPi Effect on PD-L1 Expression

PARPi’s have been found to cause increased levels of PD-L1 expression in the tumor microenvironment in vitro and in vivo [37]. To confirm that the mechanism of increased PD-L1 expression was mediated by PARP, researchers developed PARP-null cellular models, which also demonstrated substantially elevated PD-L1 expression compared to cells with intact PARP expression. In cellular and animal models of TNBC, the increase in PD-L1 was dose-dependent with PARPi exposure. Interestingly, *BRCA* downregulation or knockout did not impact PARPi effect on PD-L1 expression in TNBC [37]. Mechanistically, PARPi-induced upregulation of PD-L1 has been attributed to the STAT, NF-κB, and mammalian target of rapamycin (mTOR) pathways [67,68,69]. The downstream effects of PARPi were examined with a phosphokinase antibody array screen. Following PARPi treatment, the CHK2-p53 DNA repair pathway was the most highly activated, which is consistent with prior literature involving the mechanism of PARPi [37,70,71]. Of the proteins inactivated by PARPi, downregulation was greatest in glycogen synthase kinase 3α/β (GSK3α/β) [37]. Prior research demonstrated that GSK3β induces phosphorylation-dependent proteasome degradation of PD-L1 [72]. Therefore, PARPi inactivation of GSK3α/β in turn causes PD-L1 upregulation [37]. As evidenced by the phosphokinase antibody array, PARPi mainly activates the DNA repair axis and inhibits the PD-L1 degradation pathway [37]. As a result, combination treatment with PARPi and ICIs was suggested to circumvent the PARPi-mediated upregulation of PD-L1 via checkpoint inhibition [53].

## 6. PARPi Treatment (t) Induced AML, MDS

Preclinical modeling has shown that both PARP1 and PARP2 inhibitors demonstrate dedifferentiation in murine models with induction of T cell leukemia [45]. PARPi therapy has been associated with an increased incidence of treatment related myelodysplastic syndrome and acute myeloid leukemia [73]. In one meta-analysis of 28 randomized controlled trials involving PARPi treatment which enrolled 7306 patients, the incidence of AML and/or MDS was significantly greater (*p* = 0.026) in the PARPi cohort [74]. A separate meta-analysis supported these results with involvement of 5739 patients engaged in 14 studies, which revealed higher induction of tMDS/AML in the PARPi cohort compared to control (IRR 5.43, 95% CI 1.51–19.60) [75]. The clinical difference of tAML/MDS compared to non-treatment related AML/MDS is concerning in that 5 year survival of tAML/MDS induced by other products is less than 10% [76]. Additionally, the most common latency from time of inducible treatment to occurrence of AML/MDS is usually 5–7 years [76,77,78]. However, long term follow up in reports provided with PARPi treatment are generally less than 5 years [74]. One trial involving 130 patients receiving PARPi suggested, however, with long term follow up, the risk of tAML/MDS increased up to 6.9% beyond 5 years [23].

## 7. ICI Mechanism, Biomarkers, and Indications

PD-L1/PD-1 are currently two of the most widely studied immune checkpoints in oncology. PD-L1 functions by sending a negative signal to cytotoxic T cells resulting in apoptosis [79]. In addition, PD-L1 is involved in regulating the protein kinase B and mTOR pathway resulting in decreased glycolytic enzyme translation [80]. Upregulation of PD-L1 is a common mechanism by which cancer cells evade the immune system. PD-1 is considered protective to host cells and terminates the immune response once the malignancy has been cleared [81].

Another commonly targeted immune checkpoint is cytotoxic T-lymphocyte associated protein 4 (CTLA-4). CTLA-4 is an inhibitory checkpoint receptor that is expressed on naïve T cells during T-cell priming when T-cell receptors are engaged. CTLA-4 functions as a negative regulator by binding to B7 on APCs which disrupts the CD28-B7 co-stimulatory signal needed for T-cell activation and proliferation [82,83]. CTLA-4 inhibitors function by increasing T-cell priming and response to tumor neoantigens [81]. ICIs remove the negative regulatory signals of T-cell activation, enabling an antitumor response to recognize tumor antigens [83]. As such, treatment utilizing a PD-1/PD-L1 or CTLA-4 inhibitor will lead to activation of the immune system and an antitumor response [84].

Several biomarkers have been found to predict treatment response to ICIs, namely PD-1/PD-L1, CTLA-4, TMB, and TILs, which have been extensively described in recent literature [8,9,10,13]. Currently, there are several ICIs in clinical practice including ipilimumab, nivolumab, pembrolizumab, cemiplimab, atezolizumab, avelumab, dostarlimab, and durvalumab [81,83,85,86,87,88,89].

ICIs have also shown clinical efficacy as therapeutic agents in multiple solid tumor types and biomarkers that lead to tumor-induced immune suppression and evasion have been developed. Inflammatory cytokines and low oxygen levels in the TME upregulate PD-L1 expression on the surface of many tumor cells [90]. Binding of this transmembrane protein to its receptor, programmed cell death 1 (PD-1), suppresses the immune system by inhibiting T-cell proliferation, cytokine production, and expression of apoptosis-related genes [91,92]. Therefore, upregulated PD-L1 expression allows cancer cells to evade the host immune system and is often used as a predictor of response to ICIs that inhibit the interaction between PD-1 and PD-L1 [93]. Even so, few studies have demonstrated clinical benefit of ICIs irrespective of PD-L1 expression [94,95].

TMB is another biomarker used to predict treatment response to ICIs. It represents the number of somatic gene mutations within the DNA of malignant cells and corresponds with the degree of neoantigen expression, which flags the tumor for detection by the immune system [96,97]. Despite the direct relationship between ICI response and TMB as described in some studies, challenges with using TMB as a biomarker include lack of consensus in the definition of TMB cut-offs for patient stratification, and specific gene mutations that can influence tumor response to ICI therapy regardless of TMB status [98,99].

Mismatch repair deficient (dMMR) and microsatellite instability-high (MSI-H) tumor status have also been associated with improved ICI response. A high TMB is expected with dMMR and MSI-H tumors as a result of the defective genomic repair machinery that leads to a high mutation associated neo-antigen expression [100]. MSI-H and dMMR tumor types have also been found to selectively upregulate expression of multiple immune checkpoints, including PD-L1 [101,102].

## 8. ICI Monotherapy in Ovarian Cancer

ICI monotherapy has been explored in several clinical trials in patients with ovarian cancer. However, despite promising initial results in small clinical trials, efficacy has been limited.

The clinical efficacy and tolerability of ipilimumab, a CTLA-4 inhibitor, for patients with recurrent platinum-sensitive ovarian cancer was evaluated in a Phase II study (NCT01611558). Of the 40 patients treated, the overall response rate (ORR) was 15% [103]. Despite this, 38 patients (95%) did not complete the induction phase, and the remaining 2 patients did not complete the maintenance phase. Treatment discontinuation was mainly attributed to disease progression (*n* = 18, 45%) and drug toxicity (*n* = 15, 37.5%) [103]. Overall, twenty patients (50%) had treatment-related adverse effects (TRAEs) Grade 3 or higher.

Nivolumab was also investigated in a single-center Phase II clinical trial in patients with platinum-resistant recurrent ovarian cancer (UMIN000005714). A total of 20 patients were treated with a median follow-up period of 11.0 months. The ORR and disease control rate (DCR) was 15% and 45%, respectively [104]. Median PFS was 3.5 months (95% CI, 1.7–3.9 months), with an OS of 20 months (95% CI, 7.0 months-not reached) [104]. The most common TRAEs included increased serum AST, hypothyroidism, lymphocytopenia, decreased serum albumin, fever, increased serum ALT, maculopapular rash, arthralgia, arrhythmia, fatigue, and anemia [104]. Eight patients (40%) encountered Grade 3 or 4 TRAEs and treatment was discontinued in 2 patients due to treatment-related thyroiditis [104].

The KEYNOTE-028 (NCT02054806) is a multicohort Phase Ib clinical trial that assessed the safety and antitumor activity of pembrolizumab in 26 patients with advanced PD-L1 positive ovarian cancer. After the median follow-up duration of 15.4 months, the ORR was 11.5% and 7 patients (26.9%) had achieved stable disease [105]. The median PFS and OS were 1.9 (95% CI, 1.8–3.5) and 13.8 months (95% CI 6.7–18.8), respectively [105]. No deaths or treatment-related discontinuation occurred during this study, but a total of 19 patients (73.1%) experienced TRAEs, most commonly arthralgia, nausea, pruritis, diarrhea, and rash [105]. Elevated plasma transaminase levels were the only TRAE that was Grade 3 or higher. Despite these results seen in Phase I, a Phase II study (KEYNOTE-100, NCT02674061), of pembrolizumab in advanced recurrent ovarian cancer demonstrated reduced benefit in the overall population but demonstrated higher response rates in PD-L1 high populations [106]. Although PFS was only 2.1 months for the study population. PD-L1 expression was measured as combined positive score (CPS). ORR was 4.1% for CPS < 1, 5.7% for CPS > 1, and 10.0% for CPS ≥ 10. TRAEs were observed in 275 patients (73.1%); 74 patients (19.7%) had TRAEs Grade 3 or higher [106]. By the data cutoff date, there were 2 deaths and 5.1% discontinued treatment secondary to TRAEs.

The JAVELIN Solid Tumor study (NCT01772004) was another Phase Ib study that investigated the safety and efficacy of single-agent ICI, with avelumab given to patients with previously treated recurrent or refractory ovarian cancer. Of 125 patients treated, a complete response occurred in 1 patient (0.8%) and 11 patients (8.8%) had a partial response after a median follow-up duration of 26.6 months for an ORR of 9.6% [107]. The DCR was 52.0% and the median PFS and OS was 2.6 and 11.2 months, respectively [107]. TRAEs Grade 3 or higher occurred in 9 patients (7.2%) and 14 patients (11.2%) had adverse events (AEs) that led to death, none of which were treatment related [107]. By the data cutoff date, 120 patients had discontinued treatment due to disease progression (70.4%), AEs (12.8%), withdrawal of consent (4.8%) or for other reasons [107].

JAVELIN Ovarian 100 (NCT02718417) and JAVELIN Ovarian 200 (NCT02580058) were two open-label, three-arm, randomized, Phase III studies that failed to demonstrate significant improvement in PFS or OS with avelumab treatment alone in patients with ovarian cancer. JAVELIN Ovarian 100 (NCT02718417) enrolled 998 patients with stage III and IV epithelial ovarian, fallopian tube, or peritoneal cancer and randomly assigned patients to receive chemotherapy followed by avelumab maintenance, chemotherapy with avelumab followed by avelumab maintenance, or chemotherapy alone. Efficacy of treatment is no longer being assessed as the trial was eventually terminated for crossing prespecified futility boundaries for PFS at an interim analysis [108]. Median PFS was not estimable with the control treatment group. When compared to chemotherapy treatment alone, the hazard ratio for PFS was 1.43 in the avelumab maintenance group (95% CI 1.05–1.95, *p* = 0·99) and 1.14 in the chemotherapy and avelumab combination group (95% CI 0.83–1.56, *p* = 0.79) [108].

In JAVELIN Ovarian 200 (NCT02580058), 566 patients with platinum-resistant or platinum-refractory ovarian cancer were randomly assigned to avelumab monotherapy, pegylated liposomal doxorubicin (PLD) monotherapy, or avelumab plus PLD combination therapy. At the data cutoff, median PFS was 3.5 months in the PLD group, 1.9 months in the avelumab group, and 3.7 months in the combination group [109]. In comparison to PLD monotherapy, the hazard ratio for PFS was 1.68 with avelumab (93.1% CI 1.32 to 2.60, *p* > 0.99) and 0.78 with combination treatment (93.1% CI 0.59 to 1.24, *p* = 0.030) [109]. Median OS was 13.1 months in the PLD group, 11.8 months in the avelumab group, and 15.7 months in the combination group [109]. The hazard ratio for OS in the avelumab and combination regimens compared to PLD monotherapy was 1.14 (88.85% CI 0.95 to 1.58, *p* = 0.83) and 0.89 (88.85% CI 0.74 to 1.24, *p* = 0.21), respectively [109].

In a Phase Ia dose-escalation study (NCT01375842), 12 patients with ovarian cancer were treated with atezolizumab. After a median treatment duration of 2.8 months, only 9 patients were evaluable for objective response. Treatment with atezolizumab demonstrated a 22% ORR with 2 patients having partial response (duration of response was 8.1 and 16.6+ months) [110]. The median PFS and OS was 2.9 and 11.3 months (95% CI 1.3–5.5; and 5.5–27.7), respectively [110]. TRAEs were seen in 11 patients (91.7%), of which fatigue and pain were the most common symptoms. Aside from 2 patients (17%) having Grade 3 Aes, there were no Aes Grade 4 or higher that resulted in withdrawal from the study.

Additionally, a trial of combination atezolizumab and bevacizumab in newly diagnosed ovarian cancer patients also failed to reach the primary endpoint of PFS. A multicenter placebo-controlled double-blind randomized Phase III trial, Imagyn050/GOG (NCT03038100) enrolled 1301 patients with newly diagnosed untreated stage III and IV ovarian cancer. Patients were treated with either atezolizumab or placebo, in combination with paclitaxel, carboplatin, and bevacizumab. Median PFS in the atezolizumab group was 19.5 versus placebo of 18.4 months (HR 0.92; 95% CI, 0.79 to 1.07 *p* = 0.28) [111]. Among the PD-L1 positive population, benefit was also limited with mPFS of 20.8 versus 18.5 months in the atezolizumab versus placebo groups, respectively (HR 0.80; 95% CI, 0.65 to 0.99; *p* = 0.38) [111]. Conclusions from this trial indicate that checkpoint inhibitor therapy is not supported in newly diagnosed ovarian cancer.

The clinical efficacy of single-agent ICI treatment in ovarian cancer have not shown efficacy. Studies that specifically explore ICI monotherapy in ovarian cancer are not only limited, but overall findings in published literature thus far have been variable. Most trials involved small patient numbers and heavily pre-treated cancers that demonstrated more favorable results, whereas large Phase II and III studies have not shown efficacy. As such, ICI monotherapy may not be the ideal treatment option for ovarian cancer. Early clinical studies investigating a combinatorial approach with ICIs to improve the prognosis of ovarian carcinoma have therefore emerged in response.

## 9. PARPi and ICI Combination Therapy

Mechanistically, PARPi’s increase PD-L1 expression which enhances immune suppression [37]. PARPi also increases the number of Treg cells and enhance their suppressive function [36,59]. Several other deterrents have also been identified with combination PARPi and ICI therapy. PARPi has been shown to inhibit DC maturation and PARPi toxic effect on bone marrow function causes suppression and decreased immune activity [53,65,66]. PARPi further interferes with CD8+ T cell maturation despite upregulating the cGAS/STING pathway which was found to be defective in ovarian cancer [57]. Various clinical trials have examined the efficacy and safety of combination therapy involving PARPi and ICIs. Completed and ongoing studies are summarized in Table 2 [40].

## 10. Safety

Novel therapeutics and their unique combinations require extensive safety analysis prior to assessment of efficacy. The safety profiles of ICIs and PARPi have been examined separately and in combination [126,127]. With respect to ICIs, the incidence of AEs depends on the agent. Anti-CTLA-4 agents such as ipilimumab were among the first ICIs developed and demonstrated a 54.1% incidence of AEs that were Grade 3 or higher [128]. The incidence is dose-dependent, with a 37% prevalence of any-grade AEs in groups receiving high-dose ipilimumab versus 18% in low-dose groups [126,128,129]. The incidence of AEs is lower in anti-PD-1/anti-PD-L1 agents, with an estimated incidence of 10% for AEs Grade 3 or higher and 5–20% for any-grade toxicity [126,130]. A meta-analysis comparing the safety, efficacy, and tolerability of single agent ICIs targeting PD-1 or PD-L1 further found differences between agents, with avelumab reporting the highest safety but the lowest efficacy compared to pembrolizumab, atezolizumab, and nivolumab [131]. The most common AEs are immune-related AEs (irAEs), which arise as ICIs directly stimulate the immune system [126,132]. There is a somewhat increased incidence of irAEs in patients with prior autoimmune disease who receive ICIs, including flare-ups in rheumatoid arthritis and thyroiditis [132,133,134]. However, the benefit-risk ratio favors the use of ICIs in select patients with prior autoimmune disease due to efficacy against cancer [135].

As the use of ICIs in clinical practice has increased, there has been tremendous improvement in the early diagnosis and management of the AEs. The incidence of fatal AEs with ICIs is 0.3–1.3%, and fatal AEs tend to occur rapidly and early in the course of therapy (median time to onset of 40 days in monotherapy) [126,136]. The most common fatal AEs varied with agent: anti-CTLA-4 agents such as ipilimumab were associated with colitis, which accounted for 70% of fatal AEs. Anti-PD-L1 and anti-PD-1 agents had more varied fatal AEs with pneumonitis (33%), hepatitis (22%), and neurotoxicity (15%), accounting for the majority of fatal AEs [126].

Class effect AEs in patients receiving PARPi tend to be hematologic in nature, including Grade 3/4 neutropenia (32.9% incidence), thrombocytopenia (15.9%), and anemia (9.1%) [44,127]. These severe events contribute to a high rate of dose-reduction or discontinuation [137,138]. Although meta-analyses are still underway, it has been suggested to dose-adjust niraparib based on platelet level, and dose–response studies have not documented a significant difference in efficacy between high and lower doses of niraparib [19,138]. In contrast, dose–response effects have been demonstrated with olaparib, suggesting that the niraparib study many have been confounded by a high starting dose [19,138,139,140]. Agents vary in their predilection for hematologic AEs, with olaparib most strongly associated with neutropenia, veliparib with neutropenia and thrombocytopenia, and niraparib with neutropenia, thrombocytopenia and anemia [44,141,142]. Other AEs include fatigue and nausea/vomiting which are mainly Grade 1 and 2 but may occur in up to 70% of patients [142,143].

Data regarding safety of combination therapy with ICI and PARPi are included below.

## 11. Clinical Trials of Combination PARPi Therapy

A Phase I/II study compared the safety and efficacy of durvalumab (ICI) in combination with olaparib (PARPi) versus combination with cediranib (VEGFR inhibitor) in treating gynecologic cancers (NCT02484404). Grade 3/4 AEs included lymphopenia in both groups; anemia in the olaparib + durvalumab group; hypertension, diarrhea, pulmonary hypertension, and pulmonary embolism in the cediranib + durvalumab group. The patients receiving olaparib plus durvalumab experienced a 67% DCR, compared to a 57% DCR in cediranib plus durvalumab group. Interestingly, all of the patients who experienced disease control with PARPi + ICI treatment were *BRCA* wild-type [117]. The Phase II study, which evaluated the effectiveness of olaparib and durvalumab combination therapy in specifically treating ovarian cancer, reported an association between increased IFNγ levels and improved PFS (HR 0.37, *p* = 0.023) after treatment [118]. The overall median PFS was 3.9 months with an ORR of 14% and DCR of 71% [118].

Another Phase I/II study examined the safety and efficacy of niraparib (PARPi) and pembrolizumab (ICI) in patients with advanced TNBC or recurrent ovarian cancer (TOPACIO/Keynote-162, NCT02657889) [112]. The most common AEs requiring treatment in the study included anemia in 19% and thrombocytopenia in 9% of patients. The ORR and DCR in all patients was 25% and 68%, respectively, both of which greatly improved to 45% and 73% in the *BRCA* mutant subgroup [112]. However, further analysis using pooled data from Phase I and II trials found that the ovarian carcinoma cohorts treated with niraparib and pembrolizumab had an 18% ORR and a 65% DCR, with ORRs being similar across all biomarker subgroups which suggests treatment efficacy regardless of biomarker status [113]. Although OS data was not mature at the time of the analysis, the study reported a median PFS of 3.4 months [113].

A Phase Ib/II study of talazoparib (PARPi) combined with avelumab (ICI) evaluated the safety and efficacy of this combination treatment in patients with locally advanced, metastatic, or recurrent breast cancer (JAVELIN PARP Medley, NCT03330405). In Phase I, 3 of 12 patients had a dose-limiting reaction including neutropenia and thrombocytopenia which was likely due to combination with checkpoint inhibitor. In Phase II, the trial divided patients into cohort 2A (TNBC) or 2B (hormone receptor positive, HER2 negative, and HRD). Cohort 2B received standard-of-care hormone-based therapy prior to randomization. The second phase of the trial included 22 patients, only 3 of which were entered into cohort 2B. TRAEs occurred in 91.7% of the patients in Phase IB and in 94.7% of patients in cohort 2A, including anemia in over half of the patients, nausea, fatigue, and thrombocytopenia. Secondary outcome measures of this study, including PFS and OS, have yet to be published, but initial analysis reported a 8.3% ORR [144]. Phase Ib/II JAVELIN PARP Medley is an ongoing study, but preliminary findings have shown an acceptable safety profile and some antitumor effects of avelumab and talazoparib combination treatment. Trial patients (*n* = 34) were treated with avelumab 800 mg IV every two weeks in combination with talazoparib 1.0 mg orally once daily. In total, 3 patients in the Phase Ib trial suffered dose-limiting toxicities including Grade 3 neutropenia and Grade 3 thrombocytopenia. No treatment related deaths were experienced. A total of 12 patients in cohort 2A, those who had received 0–2 prior chemotherapy treatment regimens, were studied further. Only 1 patient achieved partial response, 6 patients had stable disease, and 5 patients experienced disease progression [144].

A Phase I/IIb study of olaparib (PARPi) with pembrolizumab (ICI) examined safety and efficacy in patients with metastatic castration-resistant prostate cancer (mCRPC) who were previously treated with docetaxel chemotherapy (KEYNOTE-365, NCT02861573). Among the 87 patients enrolled, 84 received treatment; 29 (34.5%) patients experienced Grade 3–5 TRAEs with 2 treatment-related deaths caused by a myocardial infarction and the other due to unknown causes [145]. Of all patients who were treated with olaparib and pembrolizumab, median radiographic PFS and OS were 4.3 months and 14.4 months, respectively [145]. DCR and ORR in 24 patients who had measurable disease with ≥27 weeks of follow-up were 20.8% and 8.3%, respectively [145]. The study is ongoing but treatment with olaparib in combination with pembrolizumab has demonstrated continued antitumor activity with a manageable safety profile in patients with docetaxel-pretreated mCRPC. In this trial, patients received pembrolizumab 200 mg IV every 3 weeks in combination with Olaparib 400 mg orally twice daily. Of the 41 patients that initiated treatment, 39 experienced AEs. In total, 21 patients demonstrated Grade 3–5 toxicities. Only 1 patient experienced a treatment related death. A total of 5 patients achieved a PSA response (confirmed by ≥50% decline). Median time to confirmed PSA progression was 16 weeks, median rPFS was 5 months, and median OS was 14 months [146].

A Phase I dose escalation study examined the safety and efficacy of veliparib (PARPi) and nivolumab (ICI) in combination with either carboplatin/paclitaxel or carboplatin/pemetrexed for treatment of NSCLC (NCT02944396). A total of 25 patients enrolled; 6 patients received veliparib + nivolumab + carboplatin/paclitaxel and 19 received veliparib (dose-escalated) + nivolumab + carboplatin/pemetrexed. All 25 patients (100%) who received treatment experienced at least one TRAE, of which 18 patients (72%) experienced at least one Grade 3–4 TRAEs leading to 6 patients (24%) discontinuing treatment [147]. Five patient (20%) deaths, 4 due to disease progression and 1 for unknown causes, occurred within 100 days of receiving the last treatment dose, but none were reported to be treatment-related [147]. ORR was 40.0% in the overall population, 33% in patients treated with veliparib + nivolumab + carboplatin/paclitaxel, and 42.1% in those treated with veliparib + nivolumab + carboplatin/pemetrexed [147]. These preliminary findings demonstrate the tolerable safety profile and additive antitumor activity of combination therapy with veliparib, nivolumab, and platinum double chemotherapy.

Each study reported promising results on the safety and efficacy profile of PARPi and ICI combination treatment. It also appears that AEs are consistent with those seen with individual agents and can be managed appropriately. However, most of these clinical trials are ongoing or still in its early phases using non-randomized controlled study designs with small sample sizes. Furthermore, these studies have yet to obtain sufficient follow-up time and the relevant data to conduct a specific OS analysis, which is the gold standard to reflect clinical efficacy. The different inclusion criteria used by each study and insufficient number of related studies further limit the generalization of these results to ovarian cancer. Therefore, more robust studies in ovarian cancer with long-term follow-up and biomarker analysis are needed to further elaborate on the benefits of PARPi and ICI combination therapy.

## 12. Conclusions

Precision medicine has introduced novel therapeutics to the ovarian cancer landscape, and the potential for combination therapy is an intriguing prospect for oncologists and patients alike. PARPi target the DNA repair machinery to potentiate DNA damage and disrupt cancer proliferation, whereas ICIs target the immunoinhibitory interaction between immune cells and cancer cells, thereby promoting anticancer activity of the immune system. Therefore, combined treatment with PARPi and ICIs should theoretically enhance anticancer response, particularly to cancers with high TMB and increased neoantigen expression, such as those with *BRCA1/2* mutations or HRD profiles. However, evidence supporting the clinical benefits of PARPi and ICI combination treatment is still lacking. Several trials investigating the efficacy of this combination treatment in ovarian and other non-gynecologic cancers are underway, but it is unlikely that results will be published soon. The exact mechanism of the interaction between PARPi and ICI should also be further elucidated. As clinical results continue to populate the literature, scientists should carefully examine for effective biomarkers which are predictive of sensitivity and resistance to treatment, particularly with PARPi and ICI combination therapy.

## Figures and Tables

**Table 1:** Overall survival results of PARP inhibitors in recurrent ovarian cancer.

Trial	Treatment	OS Results
SOLO3	Olaparib vs. paclitaxel, topotecan gemcitabine or pegylated liposomal doxorubicin	34.9 vs. 32.9 mos (HR 1.07 (0.76–1.49) *p* = 0.714)
Ariel4	Rucaparib vs. chemotherapy	19.4 vs. 25.4 mos (HR 1.31 (1.00–1.73) *p* = 0.0507)
NOVA	Niraparib vs. placebo	(g)*BRCA*mutated 40.9 vs. 39.1 mos (HR 0.85 (0.61–1.20) *BRCA*wt 31.0 vs. 34.8 mos (HR 1.06 (0.81–1.95)

OS, overall survival; mos, months; HR, Hazard Ratio.

**Table 2:** Clinical trials involving combination PARPi and ICI therapy have been included along with the National Clinical Trial (NCT) identifier, trial name, and phase of study, if applicable. Included studies represent the various histological cancers being treated and different drug combinations being used. Agents that are not PARPi or ICIs include prednisone (corticosteroid); cediranib and bevacizumab (VEGF inhibitors); carboplatin, paclitaxel, and pemetrexed (traditional chemotherapeutics); and enzalutamide and abiraterone acetate (hormone therapies).

Reference	Trial	Sample Size	Cancer Populations	PARPi + ICI Combination	Results
[112]	TOPACIO/Keynote-162 NCT02657889 Phase I/II	*n* = 60	Recurrent ovarian and triple negative breast cancer	Niraparib + Pembrolizumab	Overall population: ORR 25%, DCR 68% *BRCA* mutation: ORR 45%, DCR 73%
[113]		*n* = 60	Recurrent ovarian cancer		ORR 18%, DCR 65%, PFS 3.4 months
[114]		*n* = 55	Triple negative breast cancer		Overall population: ORR 21%, DCR 49% *BRCA* mutation: ORR 47%, DCR 80%, PFS 8.3 months *BRCA* wild-type: ORR 11%, DCR 33%, PFS 2.1 months
[115]	NCT02953457 Phase I/II		Recurrent or refractory ovarian, fallopian tube, or primary peritoneal cancer	Olaparib + Durvalumab + Tremelimumab	*Ongoing study*
[116]	NCT02484404 Phase I/II		Advanced or recurrent ovarian, triple negative breast, lung, prostate, and colorectal cancers	Olaparib + Durvalumab vs. Cediranib + Durvalumab	*Study Recruiting*
[117]		*n* = 19	Advanced or recurrent ovarian, triple negative breast, cervical and uterine cancers		Olaparib + Durvalumab: DCR 67% Cediranib + Durvalumab: DCR 57%
[118]		*n* = 35	Recurrent ovarian cancer	Olaparib + Durvalumab	ORR 14%, DCR 71%, PFS 3.9 months
[119]		*n* = 17	Prostate		PFS 16.1 months
[120]		*n* = 19	SCLC		ORR 10.5%, PFS 4.1 months, OS 4.1 months
[121]	NCT02571725 Phase I/II		g*BRCA*m ovarian, tubal, or primary peritoneal cancer	Olaparib + Tremelimumab	*Ongoing study*
[122]	NCT02485990		Recurrent or persistent epithelial ovarian cancer	Olaparib + Tremelimumab vs. Tremelimumab monotherapy	*Terminated*
[123]	DUO-O NCT03737643 Phase III		Advanced, high-grade epithelial ovarian cancer	*Maintenance treatment*: Bevacizumab monotherapy vs. Bevacizumab + Durvalumab vs. Bevacizumab + Durvalumab + Olaparib	*Study recruiting*
[124]	ATHENA NCT03522246 Phase III		Newly diagnosed ovarian cancer	*Maintenance treatment*: Rucaparib monotherapy vs. Nivolumab monotherapy vs. Rucaparib + Nivolumab	*Ongoing study*
[125]	ENGOT-OV44/FIRST NCT03602859 Phase III		Stage 3/4 non-mucinous epithelial ovarian, fallopian tube, or primary peritoneal cancer	*Maintenance treatment*: Bevacizumab monotherapy vs. Bevacizumab + Niraparib vs. Bevacizumab + Niraparib + Dostarlimab	*Ongoing study*

## Data Availability

No new data were created or analyzed in this study. Data sharing is not applicable to this article.

## References

[1] Staats H., Cassidy C., Kelso J., Mack S., Morand S., Choucair K., Qaqish H., Willis O., Craig D., Duff J. (2020). Targeted Molecular Therapy in Palliative Cancer Management. Insights Biomed..

[2] Bitler B.G., Watson Z.L., Wheeler L.J., Behbakht K. (2017). PARP inhibitors: Clinical utility and possibilities of overcoming resistance. Gynecol. Oncol..

[3] Franzese E., Centonze S., Diana A., Carlino F., Guerrera L.P., Di Napoli M., De Vita F., Pignata S., Ciardiello F., Orditura M. (2019). PARP inhibitors in ovarian cancer. Cancer Treat. Rev..

[4] Lightfoot M., Montemorano L., Bixel K. (2020). PARP Inhibitors in Gynecologic Cancers: What Is the Next Big Development?. Curr. Oncol. Rep..

[5] Ledermann J.A. (2016). PARP inhibitors in ovarian cancer. Ann. Oncol..

[6] Chelariu-Raicu A., Zibetti Dal Molin G., Coleman R.L. (2020). The new world of poly-(ADP)-ribose polymerase inhibitors (PARPi) used in the treatment of gynecological cancers. Int. J. Gynecol. Cancer.

[7] Liu M., Guo F. (2018). Recent updates on cancer immunotherapy. Precis. Clin. Med..

[8] Choucair K., Morand S., Stanbery L., Edelman G., Dworkin L., Nemunaitis J. (2020). TMB: A promising immune-response biomarker, and potential spearhead in advancing targeted therapy trials. Cancer Gene Ther..

[9] Keenan T.E., Burke K.P., Van Allen E.M. (2019). Genomic correlates of response to immune checkpoint blockade. Nat. Med..

[10] Conway J.R., Kofman E., Mo S.S., Elmarakeby H., Van Allen E. (2018). Genomics of response to immune checkpoint therapies for cancer: Implications for precision medicine. Genome Med..

[11] Mantia-Smaldone G.M., Corr B., Chu C.S. (2012). Immunotherapy in ovarian cancer. Hum. Vaccin. Immunother..

[12] Kandalaft L.E., Powell D.J., Singh N., Coukos G. (2011). Immunotherapy for ovarian cancer: What’s next?. J. Clin. Oncol..

[13] Morand S., Devanaboyina M., Staats H., Stanbery L., Nemunaitis J. (2021). Ovarian Cancer Immunotherapy and Personalized Medicine. Int. J. Mol. Sci..

[14] Kaufman B., Shapira-Frommer R., Schmutzler R.K., Audeh M.W., Friedlander M., Balmana J., Mitchell G., Fried G., Stemmer S.M., Hubert A. (2015). Olaparib monotherapy in patients with advanced cancer and a germline BRCA1/2 mutation. J. Clin. Oncol..

[15] Faraoni I., Graziani G. (2018). Role of BRCA Mutations in Cancer Treatment with Poly(ADP-ribose) Polymerase (PARP) Inhibitors. Cancers.

[16] Thomas A., Murai J., Pommier Y. (2018). The evolving landscape of predictive biomarkers of response to PARP inhibitors. J. Clin. Investig..

[17] Min A., Im S.A. (2020). PARP Inhibitors as Therapeutics: Beyond Modulation of PARylation. Cancers.

[18] Banerjee S., Moore K.N., Colombo N., Scambia G., Kim B.G., Oaknin A., Friedlander M., Lisyanskaya A., Floquet A., Leary A. (2021). Maintenance olaparib for patients with newly diagnosed advanced ovarian cancer and a BRCA mutation (SOLO1/GOG 3004): 5-year follow-up of a randomised, double-blind, placebo-controlled, phase 3 trial. Lancet Oncol..

[19] Gonzalez-Martin A., Pothuri B., Vergote I., DePont Christensen R., Graybill W., Mirza M.R., McCormick C., Lorusso D., Hoskins P., Freyer G. (2019). Niraparib in Patients with Newly Diagnosed Advanced Ovarian Cancer. N. Engl. J. Med..

[20] Moore K., Colombo N., Scambia G., Kim B.-G., Oaknin A., Friedlander M., Lisyanskaya A., Floquet A., Leary A., Sonke G.S. (2018). Maintenance Olaparib in Patients with Newly Diagnosed Advanced Ovarian Cancer. N. Engl. J. Med..

[21] Ray-Coquard I., Pautier P., Pignata S., Pérol D., González-Martín A., Berger R., Fujiwara K., Vergote I., Colombo N., Mäenpää J. (2019). Olaparib plus Bevacizumab as First-Line Maintenance in Ovarian Cancer. N. Engl. J. Med..

[22] Pujade-Lauraine E., Ledermann J.A., Selle F., Gebski V., Penson R.T., Oza A.M., Korach J., Huzarski T., Poveda A., Pignata S. (2017). Olaparib tablets as maintenance therapy in patients with platinum-sensitive, relapsed ovarian cancer and a BRCA1/2 mutation (SOLO2/ENGOT-Ov21): A double-blind, randomised, placebo-controlled, phase 3 trial. Lancet Oncol..

[23] Todisco E., Gigli F., Mantiero M., Sammassimo S., Pastano R., Ronchini C., Parma G., Lapresa M.T., Iori A.P., Bertolini F. (2021). Clinical presentation, diagnosis and management of therapy-related hematological disorders in women with epithelial ovarian cancer treated with chemotherapy and poly-ADP-ribose polymerase inhibitors: A single-center experience. Int. J. Cancer.

[24] Mirza M.R., Monk B.J., Herrstedt J., Oza A.M., Mahner S., Redondo A., Fabbro M., Ledermann J.A., Lorusso D., Vergote I. (2016). Niraparib Maintenance Therapy in Platinum-Sensitive, Recurrent Ovarian Cancer. N. Engl. J. Med..

[25] GlaxoSmithKline (2022). Zejula (niraparib) Important Prescribing Information for the Maintenance Treatment of Adult Patients with Non-gBRCAmut Recurrent Epithelial Ovarian, Fallopian Tube, or Primary Peritoneal Cancer Who Are in a Complete or Partial Response to Platinum-Based Chemotherapy in Second or Later Line Setting. Health Care Provider Letter (Niraparib).

[26] GlaxoSmithKline (2022). ZEJULA^®^ (niraparib) for the treatment of adult patients with advanced ovarian, fallopian tube, or primary peritoneal cancer who have been treated with 3 or more prior chemotherapy regimens is voluntarily withdrawn in the U.S. Health Care Provider Letter.

[27] Swisher E.M., Lin K.K., Oza A.M., Scott C.L., Giordano H., Sun J., Konecny G.E., Coleman R.L., Tinker A.V., O’Malley D.M. (2017). Rucaparib in relapsed, platinum-sensitive high-grade ovarian carcinoma (ARIEL2 Part 1): An international, multicentre, open-label, phase 2 trial. Lancet Oncol..

[28] Coleman R.L., Oza A.M., Lorusso D., Aghajanian C., Oaknin A., Dean A., Colombo N., Weberpals J.I., Clamp A., Scambia G. (2017). Rucaparib maintenance treatment for recurrent ovarian carcinoma after response to platinum therapy (ARIEL3): A randomised, double-blind, placebo-controlled, phase 3 trial. Lancet.

[29] Coleman R.L., Fleming G.F., Brady M.F., Swisher E.M., Steffensen K.D., Friedlander M., Okamoto A., Moore K.N., Efrat Ben-Baruch N., Werner T.L. (2019). Veliparib with First-Line Chemotherapy and as Maintenance Therapy in Ovarian Cancer. N. Engl. J. Med..

[30] AstraZeneca (2022). Lynparza (olaparib) for treatment of adult patients with deleterious or suspected deleterious germline BRCA-mutated (gBRCAm) advanced ovarian cancer who have been treated with three or more prior lines of chemotherapy is voluntarily withdrawn in the U.S. Health Care Provider Letter.

[31] Clovis Oncology I. Rubraca (rucaparib) for treatment of BRCA-mutated ovarian cancer after 2 or more chemotherapies is voluntarily withdrawn in the U.S. Health Care Provider Letter.

[32] Tew W.P., Lacchetti C., Kohn E.C. (2022). Poly(ADP-Ribose) Polymerase Inhibitors in the Management of Ovarian Cancer: ASCO Guideline Rapid Recommendation Update. J. Clin. Oncol..

[33] Nieborowska-Skorska M., Sullivan K., Dasgupta Y., Podszywalow-Bartnicka P., Hoser G., Maifrede S., Martinez E., Di Marcantonio D., Bolton-Gillespie E., Cramer-Morales K. (2017). Gene expression and mutation-guided synthetic lethality eradicates proliferating and quiescent leukemia cells. J. Clin. Investig..

[34] Bartl T., Paspalj V., Polterauer S., Grimm C. (2020). Current state and perspectives of checkpoint inhibitors in ovarian cancer treatment. Mag. Eur. Med. Oncol..

[35] Wu Z., Cui P., Tao H., Zhang S., Ma J., Liu Z., Wang J., Qian Y., Chen S., Huang Z. (2021). The Synergistic Effect of PARP Inhibitors and Immune Checkpoint Inhibitors. Clin. Med. Insights Oncol..

[36] Luo X., Nie J., Wang S., Chen Z., Chen W., Li D., Hu H., Li B. (2015). Poly(ADP-ribosyl)ation of FOXP3 Protein Mediated by PARP-1 Protein Regulates the Function of Regulatory T Cells. J. Biol. Chem..

[37] Jiao S., Xia W., Yamaguchi H., Wei Y., Chen M.K., Hsu J.M., Hsu J.L., Yu W.H., Du Y., Lee H.H. (2017). PARP Inhibitor Upregulates PD-L1 Expression and Enhances Cancer-Associated Immunosuppression. Clin. Cancer Res..

[38] Peyraud F., Italiano A. (2020). Combined PARP Inhibition and Immune Checkpoint Therapy in Solid Tumors. Cancers.

[39] Pilié P.G., Gay C.M., Byers L.A., O’Connor M.J., Yap T.A. (2019). PARP Inhibitors: Extending Benefit Beyond BRCA-Mutant Cancers. Clin. Cancer Res..

[40] Li A., Yi M., Qin S., Chu Q., Luo S., Wu K. (2019). Prospects for combining immune checkpoint blockade with PARP inhibition. J. Hematol. Oncol..

[41] Vikas P., Borcherding N., Chennamadhavuni A., Garje R. (2020). Therapeutic Potential of Combining PARP Inhibitor and Immunotherapy in Solid Tumors. Front. Oncol..

[42] Brown J.S., Sundar R., Lopez J. (2018). Combining DNA damaging therapeutics with immunotherapy: More haste, less speed. Br. J. Cancer.

[43] McGranahan N., Furness A.J., Rosenthal R., Ramskov S., Lyngaa R., Saini S.K., Jamal-Hanjani M., Wilson G.A., Birkbak N.J., Hiley C.T. (2016). Clonal neoantigens elicit T cell immunoreactivity and sensitivity to immune checkpoint blockade. Science.

[44] LaFargue C.J., Dal Molin G.Z., Sood A.K., Coleman R.L. (2019). Exploring and comparing adverse events between PARP inhibitors. Lancet Oncol..

[45] Navarro J., Gozalbo-Lopez B., Mendez A.C., Dantzer F., Schreiber V., Martinez C., Arana D.M., Farres J., Revilla-Nuin B., Bueno M.F. (2017). PARP-1/PARP-2 double deficiency in mouse T cells results in faulty immune responses and T lymphomas. Sci. Rep..

[46] de Queiroz N.M.G.P., Xia T., Konno H., Barber G.N. (2019). Ovarian Cancer Cells Commonly Exhibit Defective STING Signaling Which Affects Sensitivity to Viral Oncolysis. Mol. Cancer Res..

[47] Liang S., Hu L., Wu Z., Chen Z., Liu S., Xu X., Qian A. (2020). CDK12: A Potent Target and Biomarker for Human Cancer Therapy. Cells.

[48] Iniguez A.B., Stolte B., Wang E.J., Conway A.S., Alexe G., Dharia N.V., Kwiatkowski N., Zhang T., Abraham B.J., Mora J. (2018). EWS/FLI Confers Tumor Cell Synthetic Lethality to CDK12 Inhibition in Ewing Sarcoma. Cancer Cell.

[49] Johnson S.F., Cruz C., Greifenberg A.K., Dust S., Stover D.G., Chi D., Primack B., Cao S., Bernhardy A.J., Coulson R. (2016). CDK12 Inhibition Reverses De Novo and Acquired PARP Inhibitor Resistance in BRCA Wild-Type and Mutated Models of Triple-Negative Breast Cancer. Cell Rep..

[50] Yélamos J., Moreno-Lama L., Jimeno J., Ali S.O. (2020). Immunomodulatory Roles of PARP-1 and PARP-2: Impact on PARP-Centered Cancer Therapies. Cancers.

[51] Shou Q., Fu H., Huang X., Yang Y. (2019). PARP-1 controls NK cell recruitment to the site of viral infection. JCI Insight.

[52] Rosado M.M., Bennici E., Novelli F., Pioli C. (2013). Beyond DNA repair, the immunological role of PARP-1 and its siblings. Immunology.

[53] Lee E.K., Konstantinopoulos P.A. (2020). PARP inhibition and immune modulation: Scientific rationale and perspectives for the treatment of gynecologic cancers. Ther. Adv. Med. Oncol..

[54] Shen J., Zhao W., Ju Z., Wang L., Peng Y., Labrie M., Yap T.A., Mills G.B., Peng G. (2019). PARPi Triggers the STING-Dependent Immune Response and Enhances the Therapeutic Efficacy of Immune Checkpoint Blockade Independent of BRCAness. Cancer Res..

[55] Ivashkiv L.B., Donlin L.T. (2014). Regulation of type I interferon responses. Nat. Rev. Immunol..

[56] Zheng J., Mo J., Zhu T., Zhuo W., Yi Y., Hu S., Yin J., Zhang W., Zhou H., Liu Z. (2020). Comprehensive elaboration of the cGAS-STING signaling axis in cancer development and immunotherapy. Mol. Cancer.

[57] Pantelidou C., Sonzogni O., De Oliveria Taveira M., Mehta A.K., Kothari A., Wang D., Visal T., Li M.K., Pinto J., Castrillon J.A. (2019). PARP Inhibitor Efficacy Depends on CD8^+^ T-cell Recruitment via Intratumoral STING Pathway Activation in BRCA-Deficient Models of Triple-Negative Breast Cancer. Cancer Discov..

[58] Chabanon R.M., Muirhead G., Krastev D.B., Adam J., Morel D., Garrido M., Lamb A., Henon C., Dorvault N., Rouanne M. (2019). PARP inhibition enhances tumor cell-intrinsic immunity in ERCC1-deficient non-small cell lung cancer. J. Clin. Investig..

[59] Nasta F., Laudisi F., Sambucci M., Rosado M.M., Pioli C. (2010). Increased Foxp3+ regulatory T cells in poly(ADP-Ribose) polymerase-1 deficiency. J. Immunol..

[60] Macian F. (2005). NFAT proteins: Key regulators of T-cell development and function. Nat. Rev. Immunol..

[61] Lawrence T. (2009). The nuclear factor NF-kappaB pathway in inflammation. Cold Spring Harb. Perspect. Biol..

[62] Boyman O., Sprent J. (2012). The role of interleukin-2 during homeostasis and activation of the immune system. Nat. Rev. Immunol..

[63] Li Z., Li D., Tsun A., Li B. (2015). FOXP3+ regulatory T cells and their functional regulation. Cell. Mol. Immunol..

[64] Ma G.F., Chen S.Y., Sun Z.R., Miao Q., Liu Y.M., Zeng X.Q., Luo T.C., Ma L.L., Lian J.J., Song D.L. (2013). FoxP3 inhibits proliferation and induces apoptosis of gastric cancer cells by activating the apoptotic signaling pathway. Biochem. Biophys. Res. Commun..

[65] Echeverri Tirado L.C., Ghonim M.A., Wang J., Al-Khami A.A., Wyczechowska D., Luu H.H., Kim H., Sanchez-Pino M.D., Yelamos J., Yassin L.M. (2019). PARP-1 Is Critical for Recruitment of Dendritic Cells to the Lung in a Mouse Model of Asthma but Dispensable for Their Differentiation and Function. Mediators Inflamm..

[66] Aldinucci A., Gerlini G., Fossati S., Cipriani G., Ballerini C., Biagioli T., Pimpinelli N., Borgognoni L., Massacesi L., Moroni F. (2007). A key role for poly(ADP-ribose) polymerase-1 activity during human dendritic cell maturation. J. Immunol..

[67] Lim S.O., Li C.W., Xia W., Cha J.H., Chan L.C., Wu Y., Chang S.S., Lin W.C., Hsu J.M., Hsu Y.H. (2016). Deubiquitination and Stabilization of PD-L1 by CSN5. Cancer Cell.

[68] Bellucci R., Martin A., Bommarito D., Wang K.S., Freeman G.J., Ritz J. (2013). JAK1 and JAK2 Modulate Tumor Cell Susceptibility To Natural Killer (NK) Cells Through Regulation of PDL1 Expression. Blood.

[69] Parsa A.T., Waldron J.S., Panner A., Crane C.A., Parney I.F., Barry J.J., Cachola K.E., Murray J.C., Tihan T., Jensen M.C. (2007). Loss of tumor suppressor PTEN function increases B7-H1 expression and immunoresistance in glioma. Nat. Med..

[70] Hirao A., Kong Y.Y., Matsuoka S., Wakeham A., Ruland J., Yoshida H., Liu D., Elledge S.J., Mak T.W. (2000). DNA damage-induced activation of p53 by the checkpoint kinase Chk2. Science.

[71] Anderson V.E., Walton M.I., Eve P.D., Boxall K.J., Antoni L., Caldwell J.J., Aherne W., Pearl L.H., Oliver A.W., Collins I. (2011). CCT241533 is a potent and selective inhibitor of CHK2 that potentiates the cytotoxicity of PARP inhibitors. Cancer Res..

[72] Li C.W., Lim S.O., Xia W., Lee H.H., Chan L.C., Kuo C.W., Khoo K.H., Chang S.S., Cha J.H., Kim T. (2016). Glycosylation and stabilization of programmed death ligand-1 suppresses T-cell activity. Nat. Commun..

[73] Moore K.N. (2021). PARP inhibitor associated treatment related myeloid neoplasms: What was a “Rare” complication may be less so. Gynecol. Oncol..

[74] Morice P.-M., Leary A., Dolladille C., Chrétien B., Poulain L., González-Martín A., Moore K., O’Reilly E.M., Ray-Coquard I., Alexandre J. (2021). Myelodysplastic syndrome and acute myeloid leukaemia in patients treated with PARP inhibitors: A safety meta-analysis of randomised controlled trials and a retrospective study of the WHO pharmacovigilance database. Lancet Haematol..

[75] Nitecki R., Melamed A., Gockley A.A., Floyd J., Krause K.J., Coleman R.L., Matulonis U.A., Giordano S.H., Lu K.H., Rauh-Hain J.A. (2021). Incidence of myelodysplastic syndrome and acute myeloid leukemia in patients receiving poly-ADP ribose polymerase inhibitors for the treatment of solid tumors: A meta-analysis of randomized trials. Gynecol. Oncol..

[76] Godley L.A., Larson R.A. (2008). Therapy-related myeloid leukemia. Semin. Oncol..

[77] Kern W., Haferlach T., Schnittger S., Hiddemann W., Schoch C. (2004). Prognosis in therapy-related acute myeloid leukemia and impact of karyotype. J. Clin. Oncol..

[78] Kayser S., Dohner K., Krauter J., Kohne C.H., Horst H.A., Held G., von Lilienfeld-Toal M., Wilhelm S., Kundgen A., Gotze K. (2011). The impact of therapy-related acute myeloid leukemia (AML) on outcome in 2853 adult patients with newly diagnosed AML. Blood.

[79] Robson M., Im S.A., Senkus E., Xu B., Domchek S.M., Masuda N., Delaloge S., Li W., Tung N., Armstrong A. (2017). Olaparib for Metastatic Breast Cancer in Patients with a Germline BRCA Mutation. N. Engl. J. Med..

[80] Chang C.H., Qiu J., O’Sullivan D., Buck M.D., Noguchi T., Curtis J.D., Chen Q., Gindin M., Gubin M.M., van der Windt G.J. (2015). Metabolic Competition in the Tumor Microenvironment Is a Driver of Cancer Progression. Cell.

[81] Liu D., Jenkins R.W., Sullivan R.J. (2019). Mechanisms of Resistance to Immune Checkpoint Blockade. Am. J. Clin. Dermatol..

[82] Walker L.S., Sansom D.M. (2015). Confusing signals: Recent progress in CTLA-4 biology. Trends Immunol..

[83] Wei S.C., Duffy C.R., Allison J.P. (2018). Fundamental Mechanisms of Immune Checkpoint Blockade Therapy. Cancer Discov..

[84] Granier C., De Guillebon E., Blanc C., Roussel H., Badoual C., Colin E., Saldmann A., Gey A., Oudard S., Tartour E. (2017). Mechanisms of action and rationale for the use of checkpoint inhibitors in cancer. ESMO Open.

[85] Mok T.S.K., Wu Y.-L., Kudaba I., Kowalski D.M., Cho B.C., Turna H.Z., Castro G., Srimuninnimit V., Laktionov K.K., Bondarenko I. (2019). Pembrolizumab versus chemotherapy for previously untreated, PD-L1-expressing, locally advanced or metastatic non-small-cell lung cancer (KEYNOTE-042): A randomised, open-label, controlled, phase 3 trial. Lancet.

[86] Migden M.R., Khushalani N.I., Chang A.L.S., Lewis K.D., Schmults C.D., Hernandez-Aya L., Meier F., Schadendorf D., Guminski A., Hauschild A. (2020). Cemiplimab in locally advanced cutaneous squamous cell carcinoma: Results from an open-label, phase 2, single-arm trial. Lancet Oncol..

[87] Kim E.S. (2017). Avelumab: First Global Approval. Drugs.

[88] Syed Y.Y. (2017). Durvalumab: First Global Approval. Drugs.

[89] Narayan P., Wahby S., Gao J.J., Amiri-Kordestani L., Ibrahim A., Bloomquist E., Tang S., Xu Y., Liu J., Fu W. (2020). FDA Approval Summary: Atezolizumab Plus Paclitaxel Protein-bound for the Treatment of Patients with Advanced or Metastatic TNBC Whose Tumors Express PD-L1. Clin. Cancer Res..

[90] Zhang X., Zeng Y., Qu Q., Zhu J., Liu Z., Ning W., Zeng H., Zhang N., Du W., Chen C. (2017). PD-L1 induced by IFN-gamma from tumor-associated macrophages via the JAK/STAT3 and PI3K/AKT signaling pathways promoted progression of lung cancer. Int. J. Clin. Oncol..

[91] Hofmeyer K.A., Jeon H., Zang X. (2011). The PD-1/PD-L1 (B7-H1) pathway in chronic infection-induced cytotoxic T lymphocyte exhaustion. J. Biomed. Biotechnol..

[92] Patsoukis N., Brown J., Petkova V., Liu F., Li L., Boussiotis V.A. (2012). Selective effects of PD-1 on Akt and Ras pathways regulate molecular components of the cell cycle and inhibit T cell proliferation. Sci. Signal.

[93] Topalian S.L., Hodi F.S., Brahmer J.R., Gettinger S.N., Smith D.C., McDermott D.F., Powderly J.D., Carvajal R.D., Sosman J.A., Atkins M.B. (2012). Safety, activity, and immune correlates of anti-PD-1 antibody in cancer. N. Engl. J. Med..

[94] Carbone D.P., Reck M., Paz-Ares L., Creelan B., Horn L., Steins M., Felip E., van den Heuvel M.M., Ciuleanu T.E., Badin F. (2017). First-Line Nivolumab in Stage IV or Recurrent Non-Small-Cell Lung Cancer. N. Engl. J. Med..

[95] Motzer R.J., Escudier B., McDermott D.F., George S., Hammers H.J., Srinivas S., Tykodi S.S., Sosman J.A., Procopio G., Plimack E.R. (2015). Nivolumab versus Everolimus in Advanced Renal-Cell Carcinoma. N. Engl. J. Med..

[96] Klempner S.J., Fabrizio D., Bane S., Reinhart M., Peoples T., Ali S.M., Sokol E.S., Frampton G., Schrock A.B., Anhorn R. (2020). Tumor Mutational Burden as a Predictive Biomarker for Response to Immune Checkpoint Inhibitors: A Review of Current Evidence. Oncologist.

[97] Muniyandi P., Palaninathan V., Veeranarayanan S., Ukai T., Maekawa T., Hanajiri T., Mohamed M.S. (2020). ECM Mimetic Electrospun Porous Poly (L-lactic acid) (PLLA) Scaffolds as Potential Substrates for Cardiac Tissue Engineering. Polymers.

[98] Jardim D.L., Goodman A., de Melo Gagliato D., Kurzrock R. (2021). The Challenges of Tumor Mutational Burden as an Immunotherapy Biomarker. Cancer Cell.

[99] Goodman A.M., Kato S., Bazhenova L., Patel S.P., Frampton G.M., Miller V., Stephens P.J., Daniels G.A., Kurzrock R. (2017). Tumor Mutational Burden as an Independent Predictor of Response to Immunotherapy in Diverse Cancers. Mol. Cancer Ther..

[100] Huyghe N., Baldin P., Van den Eynde M. (2020). Immunotherapy with immune checkpoint inhibitors in colorectal cancer: What is the future beyond deficient mismatch-repair tumours?. Gastroenterol. Rep..

[101] Llosa N.J., Cruise M., Tam A., Wicks E.C., Hechenbleikner E.M., Taube J.M., Blosser R.L., Fan H., Wang H., Luber B.S. (2015). The vigorous immune microenvironment of microsatellite instable colon cancer is balanced by multiple counter-inhibitory checkpoints. Cancer Discov..

[102] Xiao Y., Freeman G.J. (2015). The microsatellite instable subset of colorectal cancer is a particularly good candidate for checkpoint blockade immunotherapy. Cancer Discov..

[103] Squibb B.-M. Phase II Study of Ipilimumab Monotherapy in Recurrent Platinum-Sensitive Ovarian Cancer. [Updated 21 August 2012]. https://ClinicalTrials.gov/show/NCT01611558.

[104] Hamanishi J., Mandai M., Ikeda T., Minami M., Kawaguchi A., Murayama T., Kanai M., Mori Y., Matsumoto S., Chikuma S. (2015). Safety and Antitumor Activity of Anti-PD-1 Antibody, Nivolumab, in Patients With Platinum-Resistant Ovarian Cancer. J. Clin. Oncol..

[105] Varga A., Piha-Paul S., Ott P.A., Mehnert J.M., Berton-Rigaud D., Morosky A., Yang P., Ruman J., Matei D. (2019). Pembrolizumab in patients with programmed death ligand 1-positive advanced ovarian cancer: Analysis of KEYNOTE-028. Gynecol. Oncol..

[106] Matulonis U.A., Shapira-Frommer R., Santin A.D., Lisyanskaya A.S., Pignata S., Vergote I., Raspagliesi F., Sonke G.S., Birrer M., Provencher D.M. (2019). Antitumor activity and safety of pembrolizumab in patients with advanced recurrent ovarian cancer: Results from the phase II KEYNOTE-100 study. Ann. Oncol..

[107] Disis M.L., Taylor M.H., Kelly K., Beck J.T., Gordon M., Moore K.M., Patel M.R., Chaves J., Park H., Mita A.C. (2019). Efficacy and Safety of Avelumab for Patients With Recurrent or Refractory Ovarian Cancer: Phase 1b Results From the JAVELIN Solid Tumor Trial. JAMA Oncol..

[108] Monk B.J., Colombo N., Oza A.M., Fujiwara K., Birrer M.J., Randall L., Poddubskaya E.V., Scambia G., Shparyk Y.V., Lim M.C. (2021). Chemotherapy with or without avelumab followed by avelumab maintenance versus chemotherapy alone in patients with previously untreated epithelial ovarian cancer (JAVELIN Ovarian 100): An open-label, randomised, phase 3 trial. Lancet Oncol..

[109] Pujade-Lauraine E., Fujiwara K., Ledermann J.A., Oza A.M., Kristeleit R., Ray-Coquard I.L., Richardson G.E., Sessa C., Yonemori K., Banerjee S. (2021). Avelumab alone or in combination with chemotherapy versus chemotherapy alone in platinum-resistant or platinum-refractory ovarian cancer (JAVELIN Ovarian 200): An open-label, three-arm, randomised, phase 3 study. Lancet Oncol..

[110] Infante J.R., Braiteh F., Emens L.A., Balmanoukian A.S., Oaknin A., Wang Y., Liu B., Molinero L., Fasso M., O’Hear C. (2016). 871P—Safety, clinical activity and biomarkers of atezolizumab (atezo) in advanced ovarian cancer (OC). Ann. Oncol..

[111] Moore K.N., Bookman M., Sehouli J., Miller A., Anderson C., Scambia G., Myers T., Taskiran C., Robison K., Maenpaa J. (2021). Atezolizumab, Bevacizumab, and Chemotherapy for Newly Diagnosed Stage III or IV Ovarian Cancer: Placebo-Controlled Randomized Phase III Trial (IMagyn050/GOG 3015/ENGOT-OV39). J. Clin. Oncol..

[112] Konstantinopoulos P.A., Waggoner S.E., Vidal G.A., Mita M.M., Fleming G.F., Holloway R.W., Van Le L., Sachdev J.C., Chapman-Davis E., Colon-Otero G. (2018). TOPACIO/Keynote-162 (NCT02657889): A phase 1/2 study of niraparib + pembrolizumab in patients (pts) with advanced triple-negative breast cancer or recurrent ovarian cancer (ROC)—Results from ROC cohort. J. Clin. Oncol..

[113] Konstantinopoulos P.A., Waggoner S., Vidal G.A., Mita M., Moroney J.W., Holloway R., Van Le L., Sachdev J.C., Chapman-Davis E., Colon-Otero G. (2019). Single-Arm Phases 1 and 2 Trial of Niraparib in Combination With Pembrolizumab in Patients With Recurrent Platinum-Resistant Ovarian Carcinoma. JAMA Oncol..

[114] Vinayak S., Tolaney S.M., Schwartzberg L., Mita M., McCann G., Tan A.R., Wahner-Hendrickson A.E., Forero A., Anders C., Wulf G.M. (2019). Open-label Clinical Trial of Niraparib Combined With Pembrolizumab for Treatment of Advanced or Metastatic Triple-Negative Breast Cancer. JAMA Oncol..

[115] Roswell Park Cancer Institute, AstraZeneca Olaparib, Durvalumab, and Tremelimumab in Treating Patients with Recurrent or Refractory Ovarian, Fallopian Tube or Primary Peritoneal Cancer with BRCA1 or BRCA2 Mutation. [Updated 29 June 2017]. https://ClinicalTrials.gov/show/NCT02953457.

[116] National Cancer Institute (NCI), National Institutes of Health Clinical Center Phase I/II Study of the Anti-Programmed Death Ligand-1 Durvalumab Antibody (MEDI4736) in Combination with Olaparib and/or Cediranib for Advanced Solid Tumors and Advanced or Recurrent Ovarian, Triple Negative Breast, Lung, Prostate and Colorectal Can. [Updated 29 June 2015]. https://ClinicalTrials.gov/show/NCT02484404.

[117] Lee J.-m., Zimmer A.D.S., Lipkowitz S., Annunziata C.M., Ho T.W., Chiou V.L., Minasian L.M., Houston N.D., Ekwede I., Kohn E.C. (2016). Phase I study of the PD-L1 inhibitor, durvalumab (MEDI4736; D) in combination with a PARP inhibitor, olaparib (O) or a VEGFR inhibitor, cediranib (C) in women’s cancers (NCT02484404). J. Clin. Oncol..

[118] Lampert E.J., Zimmer A., Padget M., Cimino-Mathews A., Nair J.R., Liu Y., Swisher E.M., Hodge J.W., Nixon A.B., Nichols E. (2020). Combination of PARP Inhibitor Olaparib, and PD-L1 Inhibitor Durvalumab, in Recurrent Ovarian Cancer: A Proof-of-Concept Phase II Study. Clin. Cancer Res..

[119] Karzai F., VanderWeele D., Madan R.A., Owens H., Cordes L.M., Hankin A., Couvillon A., Nichols E., Bilusic M., Beshiri M.L. (2018). Activity of durvalumab plus olaparib in metastatic castration-resistant prostate cancer in men with and without DNA damage repair mutations. J. Immunother. Cancer.

[120] Thomas A., Vilimas R., Trindade C., Erwin-Cohen R., Roper N., Xi L., Krishnasamy V., Levy E., Mammen A., Nichols S. (2019). Durvalumab in Combination with Olaparib in Patients with Relapsed SCLC: Results from a Phase II Study. J. Thorac. Oncol..

[121] New Mexico Cancer Care Alliance PARP-Inhibition and CTLA-4 Blockade in BRCA-Deficient Ovarian Cancer. https://ClinicalTrials.gov/show/NCT02571725.

[122] Sidney Kimmel Comprehensive Cancer Center at Johns Hopkins, AstraZeneca, MedImmune LLC Study of Tremelimumab Alone or Combined with Olaparib for Patients with Persistent EOC (Epithelial Ovarian, Fallopian Tube or Primary Peritoneal Carcinoma) [Updated 8 January 2016]. https://ClinicalTrials.gov/show/NCT02485990.

[123] AstraZeneca, European Network of Gynaecological Oncological Trial Groups (ENGOT), GOG Foundation, Inc., Myriad Genetic Laboratories, Inc. Durvalumab Treatment in Combination with Chemotherapy and Bevacizumab, Followed by Maintenance Durvalumab, Bevacizumab and Olaparib Treatment in Advanced Ovarian Cancer Patients [Updated 4 January 2019]. https://ClinicalTrials.gov/show/NCT03737643.

[124] pharmaand GmbH, Bristol-Myers Squibb, Gynecologic Oncology Group, European Network of Gynaecological Oncological Trial Groups (ENGOT), Foundation Medicine A Study in Ovarian Cancer Patients Evaluating Rucaparib and Nivolumab as Maintenance Treatment Following Response to Front-Line Platinum-Based Chemotherapy [updated 14 May 2018]. https://ClinicalTrials.gov/show/NCT03522246.

[125] Tesaro, Inc., European Network of Gynaecological Oncological Trial Groups (ENGOT) A Phase 3 Comparison of Platinum-based Therapy with TSR-042 and Niraparib versus Standard of Care (SOC) Platinum-Based Therapy as First-Line Treatment of Stage III or IV Nonmucinous Epithelial Ovarian Cancer [updated 11 October 2018]. https://ClinicalTrials.gov/show/NCT03602859.

[126] Martins F., Sofiya L., Sykiotis G.P., Lamine F., Maillard M., Fraga M., Shabafrouz K., Ribi C., Cairoli A., Guex-Crosier Y. (2019). Adverse effects of immune-checkpoint inhibitors: Epidemiology, management and surveillance. Nat. Rev. Clin. Oncol..

[127] Hou W.H., Chen S.H., Yu X. (2019). Poly-ADP ribosylation in DNA damage response and cancer therapy. Mutat. Res..

[128] Eggermont A.M., Chiarion-Sileni V., Grob J.J., Dummer R., Wolchok J.D., Schmidt H., Hamid O., Robert C., Ascierto P.A., Richards J.M. (2016). Prolonged Survival in Stage III Melanoma with Ipilimumab Adjuvant Therapy. N. Engl. J. Med..

[129] Weber J.S., Kahler K.C., Hauschild A. (2012). Management of immune-related adverse events and kinetics of response with ipilimumab. J. Clin. Oncol..

[130] Gelao L., Criscitiello C., Esposito A., Goldhirsch A., Curigliano G. (2014). Immune checkpoint blockade in cancer treatment: A double-edged sword cross-targeting the host as an “innocent bystander”. Toxins.

[131] Al-Showbaki L., Nadler M., Desnoyers A., Almugbel F., Cescon D.W., Amir E. (2020). Comparative efficacy, safety, and tolerability of immune checkpoint inhibitors (ICIs) in cancer. J. Clin. Oncol..

[132] Albrethsen M., Creeden J., Morand S., DeBiase J., Berry B., Stanbery L., Edelman G., Nemunaitis J. (2020). Relationship of hypothyroidism and immune response to pegylated IL-10/nivolumab. Immunotherapy.

[133] Xie W., Huang H., Xiao S., Fan Y., Deng X., Zhang Z. (2020). Immune checkpoint inhibitors therapies in patients with cancer and preexisting autoimmune diseases: A meta-analysis of observational studies. Autoimmun. Rev..

[134] Ramos-Casals M., Brahmer J.R., Callahan M.K., Flores-Chavez A., Keegan N., Khamashta M.A., Lambotte O., Mariette X., Prat A., Suarez-Almazor M.E. (2020). Immune-related adverse events of checkpoint inhibitors. Nat. Rev. Dis. Primers.

[135] Tang H., Zhou J., Bai C. (2021). The Efficacy and Safety of Immune Checkpoint Inhibitors in Patients With Cancer and Preexisting Autoimmune Disease. Front. Oncol..

[136] Wang D.Y., Salem J.E., Cohen J.V., Chandra S., Menzer C., Ye F., Zhao S., Das S., Beckermann K.E., Ha L. (2018). Fatal Toxic Effects Associated With Immune Checkpoint Inhibitors: A Systematic Review and Meta-analysis. JAMA Oncol..

[137] Romanová M., Klát J. (2021). Adverse events of PARP inhibitors. Ceska Gynekol..

[138] Madariaga A., Bowering V., Ahrari S., Oza A.M., Lheureux S. (2020). Manage wisely: Poly (ADP-ribose) polymerase inhibitor (PARPi) treatment and adverse events. Int. J. Gynecol. Cancer.

[139] Kaye S.B., Lubinski J., Matulonis U., Ang J.E., Gourley C., Karlan B.Y., Amnon A., Bell-McGuinn K.M., Chen L.M., Friedlander M. (2012). Phase II, open-label, randomized, multicenter study comparing the efficacy and safety of olaparib, a poly (ADP-ribose) polymerase inhibitor, and pegylated liposomal doxorubicin in patients with BRCA1 or BRCA2 mutations and recurrent ovarian cancer. J. Clin. Oncol..

[140] Audeh M.W., Carmichael J., Penson R.T., Friedlander M., Powell B., Bell-McGuinn K.M., Scott C., Weitzel J.N., Oaknin A., Loman N. (2010). Oral poly(ADP-ribose) polymerase inhibitor olaparib in patients with BRCA1 or BRCA2 mutations and recurrent ovarian cancer: A proof-of-concept trial. Lancet.

[141] Zhou J.X., Feng L.J., Zhang X. (2017). Risk of severe hematologic toxicities in cancer patients treated with PARP inhibitors: A meta-analysis of randomized controlled trials. Drug Des. Dev. Ther..

[142] Gogineni V., Morand S., Staats H., Royfman R., Devanaboyina M., Einloth K., Dever D., Stanbery L., Aaron P., Manning L. (2021). Current Ovarian Cancer Maintenance Strategies and Promising New Developments. J. Cancer.

[143] Lorusso D., Pignata S. (2018). PARPi related toxicities: Do we need more appropriate instruments to evaluate it?. Ann. Oncol..

[144] Yap T.A., Konstantinopoulos P., Telli M.L., Saraykar S., Beck J.T., Galsky M.D., Abraham J., Wise D.R., Khasraw M., Rubovszky G. (2020). Abstract P1-19-03: JAVELIN PARP Medley, a phase 1b/2 study of avelumab plus talazoparib: Results from advanced breast cancer cohorts. Cancer Res..

[145] Yu E.Y., Rodriguez J.M.M.P., Gravis G., Laguerre B., Arija J.A.A., Oudard S., Fong P.C.C., Kolinsky M.P., Augustin M., Todenhöfer T. (2020). Pembrolizumab (pembro) plus olaparib in patients (pts) with docetaxel-pretreated metastatic castration-resistant prostate cancer (mCRPC): KEYNOTE-365 cohort A efficacy, safety, and biomarker results. J. Clin. Oncol..

[146] Yu E.Y., Massard C., Retz M., Tafreshi A., Galceran J.C., Hammerer P., Fong P.C.C., Shore N.D., Joshua A., Linch M.D. (2019). Keynote-365 cohort a: Pembrolizumab (pembro) plus olaparib in docetaxel-pretreated patients (pts) with metastatic castrate-resistant prostate cancer (mCRPC). J. Clin. Oncol..

[147] Clarke J.M., Patel J.D., Robert F., Kio E.A., Thara E., Ross Camidge D., Dunbar M., Nuthalapati S., Dinh M.H., Bach B.A. (2021). Veliparib and nivolumab in combination with platinum doublet chemotherapy in patients with metastatic or advanced non-small cell lung cancer: A phase 1 dose escalation study. Lung Cancer.

